# A Critical Domain of Ebolavirus Envelope Glycoprotein Determines Glycoform and Infectivity

**DOI:** 10.1038/s41598-018-23357-8

**Published:** 2018-04-03

**Authors:** Haruhiko Fujihira, Katsuaki Usami, Keita Matsuno, Hideyuki Takeuchi, Kaori Denda-Nagai, Jun-ichi Furukawa, Yasuro Shinohara, Ayato Takada, Yoshihiro Kawaoka, Tatsuro Irimura

**Affiliations:** 1 Laboratory of Cancer Biology and Molecular Immunology, Graduate School of Pharmaceutical Sciences, The University of Tokyo, Tokyo, 113-0033 Japan; 2 Division of Glycobiologics, Intractable Disease Research Center, Juntendo University Graduate School of Medicine, Tokyo, 113-8421 Japan; 3 Glycometabolome Team, Systems Glycobiology Research Group, Global Research Cluster, RIKEN, Saitama, 351-0198 Japan; 4 Laboratory of Microbiology, Faculty of Veterinary Medicine, Hokkaido University, Sapporo, 060-0818 Japan; 5 Division of International Services, Global Institution for Collaborative Research and Education (GI-CoRE), Hokkaido University, Sapporo, 001-0020 Japan; 6 Department of Molecular Biochemistry, Nagoya University School of Medicine, Nagoya, 4668550 Japan; 7 Laboratory of Medical and Functional Glycomics, Graduate School of Advanced Life Science, Hokkaido University, Sapporo, 001-0021 Japan; 8 Department of Advanced clinical glycobiology, Faculty of Medicine and Graduate School of Medicine, Hokkaido University, Sapporo, 001-0021 Japan; 9 Department of Pharmacy, Kinjo Gakuin University, Nagoya, 4638521 Japan; 10 Division of Global Epidemiology, Hokkaido University Research Center for Zoonosis Control, Sapporo, 001-0020 Japan; 11 CREST, Japan Science and Technology Agency, Saitama, 332-0012 Japan; 12 Division of Virology, Department of Microbiology and Immunology, Institute of Medical Science, The University of Tokyo, Tokyo, 108-8639 Japan; 13 Department of Pathobiological Sciences, University of Wisconsin, Madison, WI 53706 USA

## Abstract

Ebolaviruses comprises 5 species that exert varying degrees of mortality/infectivity in humans with Reston ebolaviruses (REBOV) showing the lowest and Zaire ebolaviruses (ZEBOV) showing the highest. However, the molecular basis of this differential mortality/infectivity remains unclear. Here, we report that the structural features of ebolavirus envelope glycoproteins (GPs) and one of their counter receptors, macrophage galactose-type calcium-type lectin (MGL/CD301), play crucial roles in determining viral infectivity. The low infectivity of REBOV mediated by the interaction between GPs and MGL/CD301 dramatically increased when the N-terminal 18 amino acids (33rd through 50th) of GPs were replaced with that of ZEBOV. Furthermore, structural analysis of glycans of GPs revealed that *N*-glycans were more extended in REBOV than in ZEBOV. *N*-glycan extension was reversed by the replacement of aforementioned N-terminal 18 amino acid residues. Therefore, these data strongly suggest that extended *N*-glycans on GPs reduce MGL/CD301-mediated viral infectivity by hindering the interaction between GPs and MGL/CD301 preferentially binds *O*-glycans.

## Introduction

Involvement of glycan-lectin binding in the initiation of virus-host interactions is widely known, yet the structural basis of the interactions is not fully understood. Lectins on the surfaces of viruses such as influenza virus, and surface glycans such as those present on ebolavirus (EBOV), are supposed to play significant roles. EBOV belongs to the family *Filoviridae* and causes severe hemorrhagic fevers in humans and nonhuman primates^1–3^. The mechanism of pathogenesis of EBOV infection remains mostly unknown, making it paramount to understand the mechanism of infection, develop effective therapies and solve the biological questions why and how such virulent viral disease gave rise. Since the pathogenesis of EBOV involves inflammation and vascular stability, the glycan-lectin interactions taking place on the surfaces of macrophages and dendritic cells are likely to play crucial roles.

It is known that the degree of pathogenicity depends on the virus species, with 5 species (*Zaire ebolavirus, Sudan ebolavirus, Bundibugyo ebolavirus, Tai Forest ebolavirus*, and *Reston ebolavirus*) identified to date. Ebolaviruses belonging to *Reston ebolavirus* (REBOV) do no cause symptomatic disease, whereas those belonging to *Zaire ebolavirus* (ZEBOV) show the highest mortality in humans^1,3^. However, the molecular basis for the differential virulence between REBOV and ZEBOV has not been fully elucidated. Although it appears that a variety of mechanisms collectively modulate the processes leading to the establishment of infection and pathogenesis^4^, the EBOV envelope glycoproteins (GPs) are known to function as one of the crucial factors that determine the differential virulence.

GPs are type I transmembrane glycoproteins composed of GP1 and GP2. GP1 has a mucin-like motif, highly modified with *N*- and *O*-glycans, and GP2 has a transmembrane region tethering the GP1-GP2 complex on the viral surface^2,3^. During the course of infection, GPs are apparently involved in viral attachment and fusion of viral envelope with host membranes, leading to the subsequent entry into cells^2,5,6^. Recent findings that GP-specific neutralizing antibodies and pseudosaccharides, which could be a ligand of lectins expressed on host cell surfaces, were reported to protect experimental animals from lethal EBOV infection also support the notion that GPs function as crucial elements in determining the degree of infection^7–10^. Additionally, the mucin-like domain of ZEBOV GP1 was previously reported to be essential to the infectivity of ZEBOV^11^.

C-Type lectins on myeloid and other cells were previously reported to act as entry sites for EBOV through their interaction with GPs^12–18^. As examples, hepatic asialoglycoprotein receptor was reported to interact with *N*-glycans promoting viral entry into hepatocytes^14^, and dendritic cell-specific ICAM3 (intercellular adhesion molecule 3)-grabbing non-integrin (DC-SIGN) and DC-SIGN-related receptor (DC-SIGNR) were shown to bind high mannose-type *N*-glycans to promote entry into dendritic cells^12,13,16,17^. Endothelial LSECtin (liver and lymph node sinusoidal endothelial cell C-type lectin) recognizes *N*-glycans and enhances viral infection^15^. We previously showed that K562 cells became susceptible to infection by vesicular stomatitis virus that lacks native G protein (VSVΔG*, hereafter described as VSV)^5^ pseudotyped with EBOV GPs when macrophage galactose-type calcium-type lectin (MGL/CD301) was expressed^18^. Since MGL/CD301 preferentially binds *O*-glycans, mucin-like domain was thought to play important roles in the interaction.

In the present report, we aimed to answer the question why the lethality/pathogenicity of REBOV is lower than that of ZEBOV. To this end, we prepared pseudotyped viruses and virus-like particles (VLPs)^19,20^ with ZEBOV GP (ZGP), REBOV GP (RGP), or chimeric GPs using HEK293T cells and compared these GPs with respect to: (1) their binding to recombinant MGL/CD301, (2) the roles of *N*-glycans and *O*-glycans of GPs on the binding to recombinant MGL/CD301, and (3) the structures of *N*-glycans present on the GPs. Our exhaustive structural analysis demonstrated that RGP’s *N*-glycans are more extended than ZGP’s *N*-glycans. When *N*-glycans were removed from GPs, the difference in the binding capacity to MGL/CD301 observed between RGP and ZGP diminished. It is likely that preferential binding of MGL/CD301 to *O*-glycans on GPs is interfered by the presence of extended *N*-glycans. By preparing chimeric GPs between RGP and ZGP, we found that the structural characteristics of RGP and ZGP were biosynthetically determined by a surprisingly short stretch of amino acid residues irrelevant to major glycosylation sites. Our results clearly showed that this short stretch of amino acid residues of GP controls the extension of *N*-glycans of the entire GP and, in turn, the degree of viral infectivity by modulating the interaction of GPs with MGL/CD301.

## Results

### MGL/CD301 plays an important role in the infection of VSV pseudotyped with RGP and ZGP into monocyte-derived immature dendritic cells

**Figure 1:**
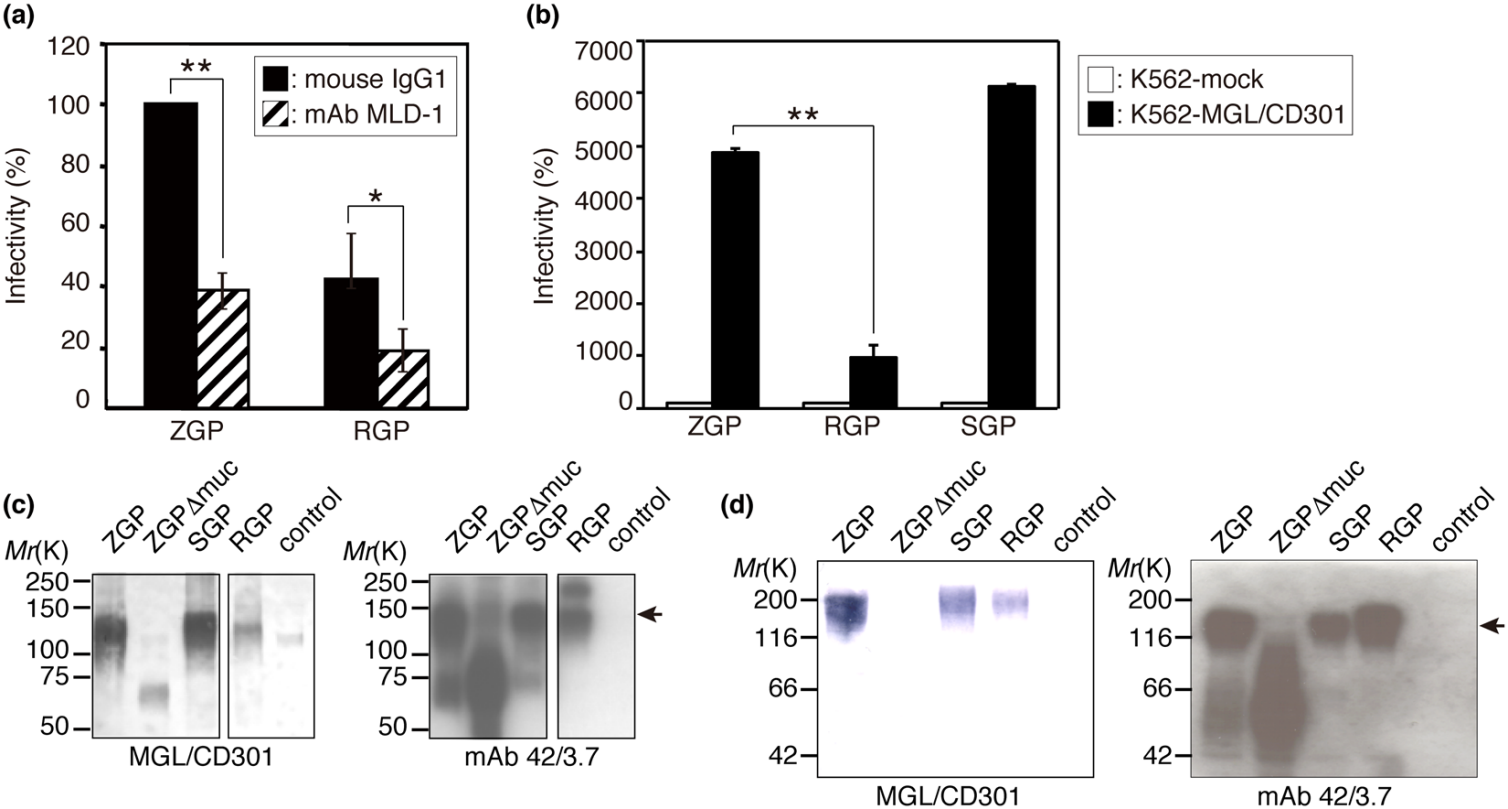
A positive correlation was found between the infectivity of VSV pseudotyped with EBOV GPs and MGL/CD301 binding to the GPs. (**a**) Blockade of infection of VSV pseudotyped with ZGP or RGP to monocyte-derived dendritic cells by a monoclonal antibody specific for MGL1/CD301 (mAb MLD-1). In this calculation, the number of infected cells with VSV pseudotyped with ZGP treated with mouse IgG1 was set to 100%, and each relative infectivity was compared by the ratio to this value. For statistical analysis, Student’s *t*-test was performed. *p < 0.05, **p < 0.01. (**b**) Effect of MGL/CD301 expression on the infection of VSV pseudotyped with ZGP, SGP, or RGP. All experiments were performed 3 times, and representative results are shown. In this calculation, the number of infected K562-mock cells with VSV pseudotyped with ZGP, RGP, or SGP was set to 100%, respectively, and the relative infectivity of K562-MGL/CD301 was shown by the ratio to this value. For statistical analysis, Student’s *t*-test was performed. *p < 0.05, **p < 0.01. (**c**) Comparison of the binding of recombinant MGL/CD301 (left panel) or anti-EBOV GP1 mAb 42/3.7 (right panel) to electrophoretically separated lysates of purified viral particles from VSV pseudotyped with ZGP, ZGP Δmuc, SGP, RGP, or control (VSVG). (**d**) Comparison of the binding of recombinant MGL/CD301 (left panel) or anti-EBOV GP1 mAb 42/3.7 (right panel) to electrophoretically separated lysates of purified VLPs with ZGP, ZGP Δmuc, SGP, RGP, or control (VSVG). Arrows in (**c**) and (**d**) indicate the position of intact GP1. For (**c**) and (**d**), cropped images from representative results are shown, and whole blot images are shown in Fig. S5. VSVG: G glycoprotein of VSV.

Attachment of EBOV to host cells is known to be mediated by the so-called mucin-like domain of GPs, with surface lectins of hepatocytes, dendritic cells, macrophages, and endothelial cells apparently being engaged with viral attachment and subsequent viral entry, as stated above. Among several C-type lectins expressed on the surfaces of dendritic cells and macrophages, a predominant role was predicted for MGL/CD301 based on our previous findings showing that infection of VSV pseudotyped with ZGP was significantly enhanced in K562 cells expressing MGL/CD301 (K562-MGL/CD301 cells)^18^. We report here that a specific blocking monoclonal antibody against MGL/CD301, mAb MLD-1^21^, significantly reduced the infectivity of VSV pseudotyped with ZGP or RGP in human monocyte-derived immature DCs. As shown in Fig. 1a, these pseudotyped viruses infected human monocyte-derived immature DCs, with VSV pseudotyped with RGP being less infective than VSV pseudotyped with ZGP. Pre-incubation of immature DCs with mAb MLD-1 reduced the infection rate to ~40% compared to an isotype control antibody (Fig. 1a). According to these results, we judged MGL/CD301 on the surface of immature DCs plays a predominant role in infection of these cells with EBOV. The results also indicate that structural characteristics of EBOV GPs determine the differential viral infectivity, because the only difference between VSV pseudotyped with RGP and VSV pseudotyped with ZGP lies in the structures of their GPs. Therefore, we focused on MGL/CD301 and GPs to explore the molecular basis of the differential infectivity between REBOV and ZEBOV.

### Infectivity of VSV pseudotyped with GPs correlates with the binding of MGL/CD301 to the GPs from VLPs

MGL/CD301-mediated infection of VSV pseudotyped with GPs (derived from ZEBOV, *Sudan ebolavirus* (SEBOV) and REBOV) was examined using K562-MGL/CD301 cells. All of the pseudotyped viruses infected K562-MGL/CD301 cells more efficiently than K562-mock cells (Fig. 1b). VSV pseudotyped with ZGP or SEBOV GP (SGP) showed significantly higher infectivity to cells expressing MGL/CD301 than VSV pseudotyped with RGP (Fig. 1b). Again, these results strongly suggest that the structural features of GPs determine the degree of the infection through their binding to MGL/CD301. To investigate this correlation further, the binding of recombinant MGL/CD301 to electrophoretically separated viral proteins of VSV pseudotyped with ZGP, ZGP lacking the mucin-like domain (Δmuc), SGP, or RGP was compared by immunoblotting. As shown in Fig. 1c, MGL/CD301 could bind to ZGP/SGP much stronger than ZGP Δmuc/RGP, whereas similar differences were not detected with the binding of anti-GP1 mAb 42/3.7, which recognizes a common epitope in RGP and ZGP^22^. The relative binding capacity of MGL/CD301 to these GPs correlated with the infectivity of pseudotyped viruses (Fig. 1b,c) in K562-MGL/CD301 cells. To further investigate the structural basis for the MGL/CD301-EBOV GP interactions, VLPs produced by HEK293T cells were used^19,23,24^. Similar to the results of the immunoblotting analysis with MGL/CD301 or mAb 42/3.7 using VSV pseudotyped with GPs, the binding of MGL/CD301 to VLP-ZGP was higher than to VLP-RGP or VLP-ZGPΔmuc (Fig. 1d). Taken together, the binding intensity of MGL/CD301 to GPs from VLPs correlated with the infectivity of pseudotyped viruses.

### The primary amino acid sequence of the so-called mucin-like domain of ZGP is not responsible for the differential infectivity and MGL/CD301 binding

**Figure 2:**
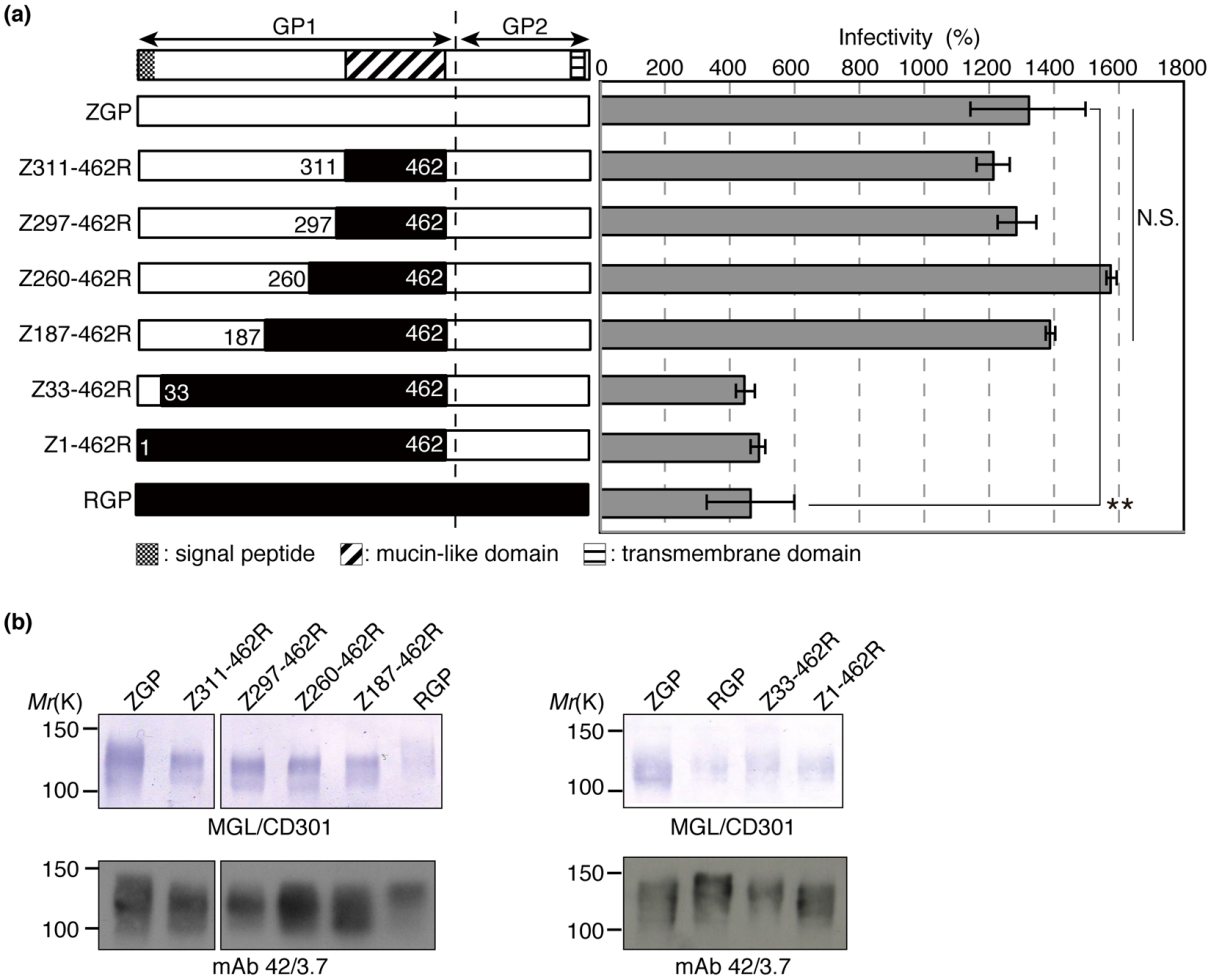
High infectivity of VSV pseudotyped with GPs and high levels of MGL/CD301 binding to the GPs depend on the 33–186 amino acid sequence of ZGP. (**a**) Diagrams showing full-length ZGP (white bar), RGP (black bar), and chimeric GPs (black and white combination bar) on the left and relative infectivity in grey bars on the right. In the chimeric GPs, the positions in the amino acid sequence of ZPG replaced with those of RGP are indicated (Z311-462R, Z297-462R, Z260-462R, Z187-462R, Z33-462R, and Z1-462R). The infectivity of VSV pseudotyped with the indicated GPs was determined using K562-MGL/CD301 cells as described in Materials and methods. All experiments were performed in triplicates, and the data shown are means ± SD. For statistical analysis, Student’s *t*-test was performed. *p < 0.05, **p < 0.01. (**b**) Lysates of VLPs were electrophoretically separated and subjected to immunoblotting using MGL/CD301 (upper panels) or mAb 42/3.7 (lower panels). Cropped images from representative results are shown and whole blot images are shown in Fig. S5.

The EBOV GP mucin-like domain (amino acid residues 311–462 of GP1) is rich in both *N*- and *O*-glycans^3^. The primary amino acid sequences of this domain are distinct between ZGP and RGP. The amino acid sequence homology between full-length ZGP and RGP is 58.1%, whereas that of the mucin-like domain is only 16.7% (see Fig. S1 for amino acid sequences). Thus, we thought that the primary amino acid sequence of the mucin-like domain determines the differential viral infectivity and differential MGL/CD301 binding. To test this hypothesis, we generated both VSV and VLP bearing a chimeric GP in which the mucin-like domain of ZGP was replaced with that of RGP. Unexpectedly, the infectivity of VSV pseudotyped with the chimeric GP was similar to that of VSV pseudotyped with ZGP in K562-MGL/CD301 cells (Fig. 2a; Z311-462R). Also, the binding of MGL/CD301 to chimeric GP from VLP was similar to that of ZGP from VLP (Fig. 2b; Z311-462R). These results indicate that the primary structure of the mucin-like domain is not responsible for the differences in MGL/CD301-mediated infectivity or the differential binding capacity to MGL/CD301 between ZGP and RGP. We could not test chimeric RGP carrying the mucin-like domain of ZGP, because the production of both pseudotyped VSV and VLP containing this domain was inefficient for unknown reasons, although the presence of this glycoprotein was confirmed on the cell surface of transfected HEK293T cells. Considering that Ser/Thr residues, representing potential *O*-glycosylation sites, are also present outside of the mucin-like domain, various lengths of polypeptide stretches around the mucin-like domain of ZGP were replaced with the corresponding region of RGP based on amino-acid sequence homology (Fig. 2a). Such chimeric GPs were examined for viral infectivity in K562-MGL/CD301 cells and MGL/CD301’s binding capacity to GPs. Even when 276 amino acids (amino acids 187 to 462) including the mucin-like domain (151 amino acids) of ZGP were replaced with the corresponding region of RGP, the infectivity of VSV pseudotyped with these chimeric GPs was very similar to that of VSV pseudotyped with ZGP (Fig. 2a). Similarly, the binding capacity of MGL/CD301 to these chimeric GPs was almost identical to the binding capacity to ZGP (Fig. 2b). However, to our surprise, replacement of amino acid residues 1–462 or 33–462 from ZGP into RGP dramatically reduced both the infectivity to K562-MGL/CD301 cells and the binding capacity to MGL/CD301 compared to ZGP (Fig. 2a,b). These results made us hypothesize that the amino acid residues 33–186 of GP might regulate the infectivity to K562-MGL/CD301 cells and the binding capacity to MGL/CD301.

### The amino acid residues 33–50 of ZGP and RGP are critical to the differential infectivity and the differential MGL/CD301 binding between ZEBOV and REBOV

**Figure 3:**
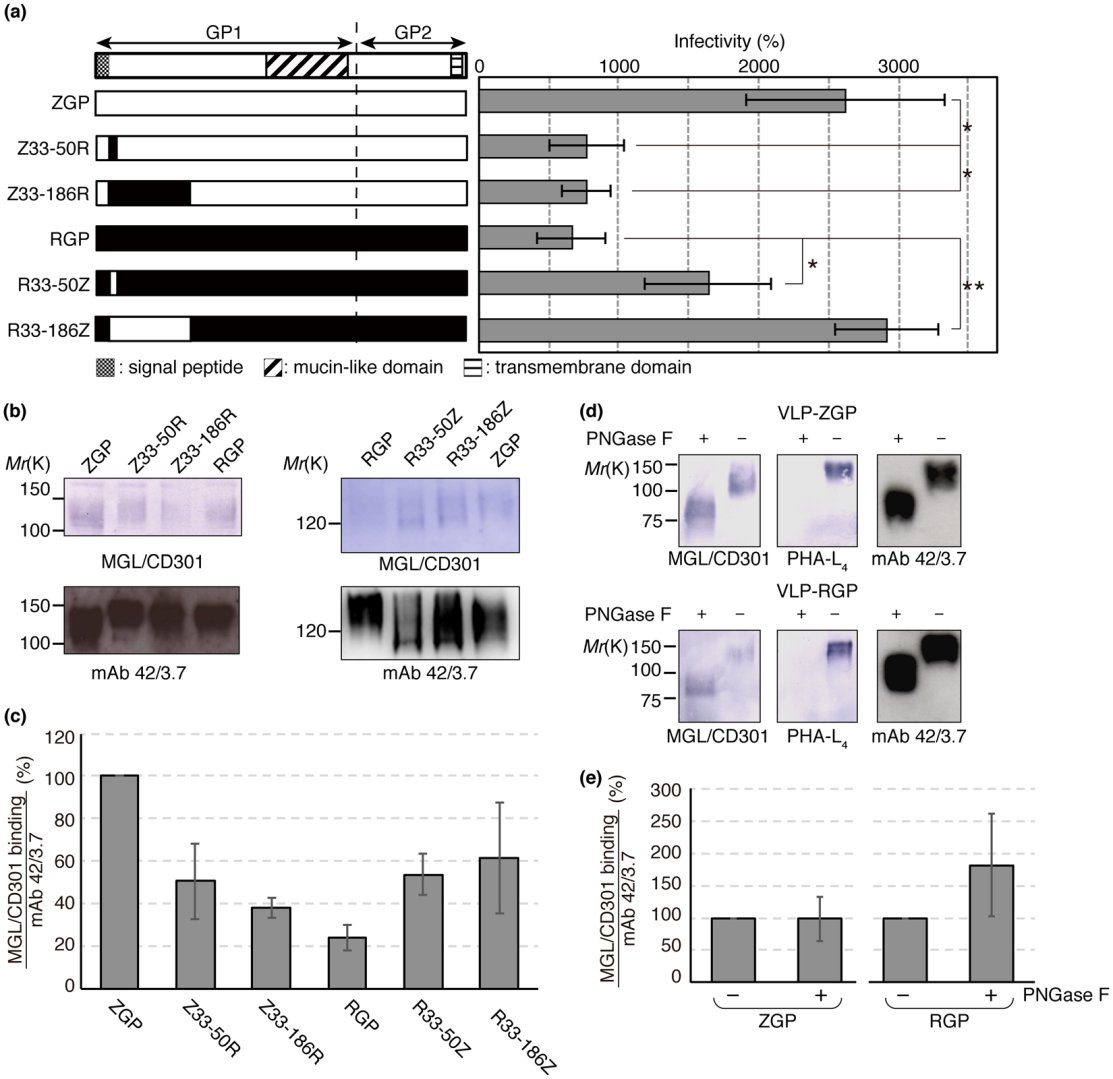
Replacement of the *N*-terminal regions of ZGP and RGP reverse the relative viral infectivity and MGL/CD301 binding. (**a**) Diagram showing ZGP (white bar), RGP (black bar), chimeric GPs (black and white combination bar), chimeric GPs at position 33–186 (Z33-186R and R33-186Z), and chimeric GPs at position 33–50 (Z33-50R and R33-50Z) used in the present study. The infectivity of VSV pseudotyped with the indicated GPs was determined using K562-MGL/CD301 cells as described in the Material and methods. Data shown are means ± SD of triplicate experiments. For statistical analysis, Student’s *t*-test was performed. *p < 0.05, **p < 0.01 (**b**,**c**) Lysates of VLPs with the indicated GPs were subjected to immunoblotting using MGL/CD301 (upper panels) or mAb 42/3.7 (lower panels). Quantification results of immunoblotting are shown in (**c**) (n = 3). In this quantification, peak areas of each band were measured and the relative ratio of MGL/CD301 to mAb 42/3.7 was calculated. The relative ratio of ZGP was set to 100%. (**d**,**e**) VLPs with ZGP or RGP were treated or untreated with PNGase F. Lysates of treated VLPs were electrophoretically separated and subjected to immunoblotting using MGL/CD301, PHA-L_4_ or mAb 42/3.7. Quantification results of immunoblotting are shown in (**e**) (n = 4). In this quantification, peak areas of each band were measured and the relative ratio of MGL/CD301 to mAb 42/3.7 was calculated. The relative ratio of ZGP/RGP without PNGase F treatment was set to 100%. The equations used for this calculation were as follow; Relative ratio of MGL/CD301 to mAb 42/3.7 of ZGP = ZGP with PNGase F treatment/ZGP without PNGase F treatment x 100 (%), Relative ratio of MGL/CD301 to mAb 42/3.7 of RGP = RGP with PNGase F treatment/RGP without PNGase F treatment x 100 (%). For (**b**) and (**d**), cropped images from representative results are shown and whole blot images are shown in Fig. S5.

To further investigate the role of amino acid residues 33–186 of GP on viral infectivity and binding capacity to MGL/CD301, we generated VSVs pseudotyped with chimeric ZGP/RGP and VLPs, both containing the swapped 33–186 amino acid residues of RGP/ZGP (Fig. 3a). Then, we examined the viral infectivity in K562-MGL/CD301 cells and the binding capacity to MGL/CD301. The infectivity of VSV pseudotyped with chimeric ZGP (ZGP 33–186 swapped into RGP) was dramatically reduced compared to ZGP (Fig. 3a; Z33-186R). Similarly, the binding capacity of MGL/CD301 to the chimeric GP was dramatically reduced compared to ZGP (Fig. 3b,c; Z33-186R). On the contrary, the infectivity of VSV pseudotyped with chimeric RGP (RGP 33–186 swapped into ZGP), and the binding capacity of MGL/CD301 to chimeric RGP were dramatically increased compared to RGP (Fig. 3a–c; R33-186Z). These results suggest that the residues 33–186 of GP have a pivotal role in determining the differential infectivity and the differential binding to MGL/CD301 between ZEBOV and REBOV. To further investigate this issue, we focused on residues 33–50, because among residues 33–186 the homology of residues 33–50 is relatively low between ZGP and RGP. By swapping residues 33–50 of ZGP into RGP, both the infectivity to K562-MGL/CD301 cells and the binding capacity to MGL/CD301 were dramatically reduced to the level of RGP (Fig. 3a–c; Z33-50R). As we expected, the infectivity of VSV pseudotyped with chimeric GP containing swapped residues 33–50 from RGP to ZGP was significantly greater than that of VSV pseudotyped with RGP (Fig. 3a; R33-50Z). Similarly, the binding capacity of MGL/CD301 to the chimeric GPs was dramatically increased compared with RGP (Fig. 3b,c; R33-50Z). These results strongly suggest that the amino acid residues 33–50 near the *N*-terminus of GP are responsible for the biosynthesis of the GP structure that determines the interactions with MGL/CD301 and, in turn, viral infectivity in K562-MGL/CD301 cells.

### Binding of EBOV GPs to MGL/CD301 is mediated by *O*-glycans, while *N*-glycans in RGP interfere with the interaction between MGL/CD301 and *O*-glycans

To examine whether *N*-glycans or *O*-glycans of RGP and ZGP determine the interaction with MGL/CD301, GPs were treated with Peptide:*N*-glycanase F (PNGase F), and the binding of MGL/CD301 was examined. Removal of *N*-glycans was confirmed by the loss of PHA-L_4_ binding and by an alteration in electrophoretic migration distance. As shown in Fig. 3d,e, the binding of MGL/CD301 to ZGP did not change after PNGase F treatment. In contrast, the binding of MGL/CD301 to RGP increased after PNGase F treatment. These results suggest that the binding of MGL/CD301 to GPs was mediated by *O*-glycans not by *N*-glycans. Additionally, the binding of MGL/CD301 to ZGP/RGP became almost identical after PNGase F treatment, suggesting that *N*-glycans in RGP interfered with the interaction between MGL/CD301 and RGP.

### RGP has more extended *N*-glycans than ZGP, and the extended *N*-glycans seem to inhibit the interaction between GP and MGL/CD301

**Figure 4:**
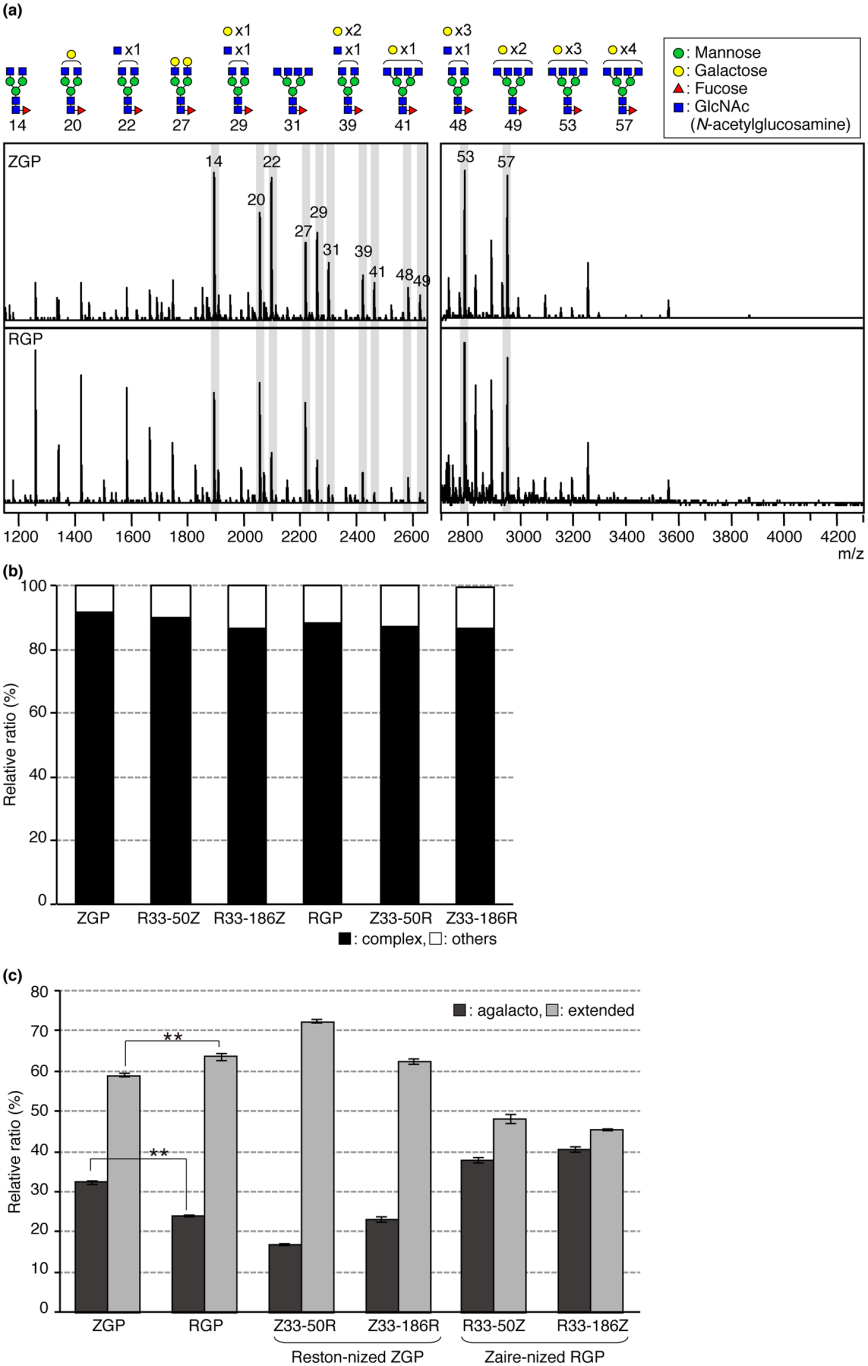
MALDI-TOF MS analyses revealed that extension patterns of *N*-glycans differ between ZGP and RGP. (**a**) Spectra of *N*-Glycans released from VLPs bearing ZGP (upper spectrum) and RGP (lower spectrum) by PNGase F treatment. Structures of glycans were estimated by mass number and MALDI-TOF/TOF analyses, and part of the estimated structures were shown on the top of the panel. The numbers written below the glycan symbols corresponded to the numbers shown in the MS spectra. For complete mass analysis data, see Table 1. MS analyses were performed three times and representative spectra are shown. (**b**) Relative quantities of complex-type and other types of *N*-glycans among all detected *N*-glycans from ZGP and RGP. Peak areas of *N*-glycans were measured by Flex analysis (Bruker), and the sum of all detected *N*-glycan peak areas from ZGP, RGP, or chimeric GPs was defined as 100%. The relative ratios of complex-type and other types of *N*-glycans were calculated. (**c**) Relative amounts of extended-type *N*-glycans and agalacto-type *N*-glycans from ZGP, RGP, or chimeric GPs were calculated in the same way as in (**b**). For statistical analysis, Student’s *t*-test was performed. **p < 0.01.

To compare the structural features of *N*-glycans present on RGP and ZGP, the structures of *N*-glycans released from RGP, ZGP, and chimeric GPs were analyzed. GPs were immunoprecipitated from dissolved VLPs using mAb 42/3.7. Immunoprecipitated GPs were subjected to in-gel PNGase F digestion followed by glycoblotting^25^ and MALDI-TOF MS. Typical MS spectra of ZGP and RGP are shown in Fig. 4a (see Fig. S2 for other GPs), and the structural features estimated from the mass numbers and TOF/TOF analysis are shown in Fig. 4a (only a part of the complex type *N*-glycans are shown in symbols). The complete estimated compositions of *N*-glycans observed in MS analyses are shown in Table 1. The structures of the detected *N*-glycans were similar between RGP and ZGP with about 90% of them being complex-type *N*-glycans (Fig. 4b). In this calculation, we analyzed the MS peak area of all detected *N*-glycans (Table 1) and defined their sum as 100%. Based on the results of our MS analyses, we then focused on complex-type *N*-glycans on GPs. Comparison of the extension pattern of complex-type *N*-glycans between RGP and ZGP revealed that there were more extended complex-type *N*-glycans on RGP than ZGP (Fig. 4c). At the same time, the amount of non-extended complex-type *N*-glycans (agalacto-type glycans) in RGP decreased (Fig. 4c). For this comparison, we defined glycan 10, 14, 16, 22, 25, and 31 in Table 1 as non-extended complex-type *N*-glycans (*i.e*. agalacto-type *N*-glycans; *N*-glycans, which have only *N*-acetylglucosamine (GlcNAc) residues at the non-reducing terminal) and we difined galactosylated and sialylated *N*-glycans (such as glycan 20, 27, 44, 55 in Table 1 in case of bi-antennary *N*-glycans) as extended complex-type *N*-glycans (*i.e*. extended-type *N*-glycans). When *N*-glycans are maturated/elongated, their structures are known to become bulky and the steric hindrance around *O*-glycans are likely to increase. Therefore, we hypothesized that the steric hindrance of extended *N*-glycans of RGP inhibit the interaction between GP and MGL/CD301, and that *N*-terminal amino acid residues 33–50 or 33–186 of GP regulate the extension of *N*-glycans.

**Table 1:** Assigned signals of oligosaccharides released from GPs.

No.	m/z	Composition
1	1178.50	(Hex)_2_(HexNAc)_2_
2	1340.55	(Hex)_3_(HexNAc)_2_
3	1486.61	(Hex)_3_(HexNAc)_2_(Fuc)_1_
4	1502.60	(Hex)_4_(HexNAc)_2_
5	1543.63	(HexNAc)_1_ + (Man)_3_(GlcNAc)_2_
6	1648.66	(Hex)_4_(HexNAc)_2_(Fuc)_1_
7	1664.65	(Hex)_2_ + (Man)_3_(GlcNAc)_2_
8	1689.69	(HexNAc)_1_(Fuc)_1_ + (Man)_3_(GlcNAc)_2_
9	1705.68	(Hex)_1_(HexNAc)_1_ + (Man)_3_(GlcNAc)_2_
10	1746.71	(HexNAc)_2_ + (Man)_3_(GlcNAc)_2_
11	1826.71	(Hex)_3_ + (Man)_3_(GlcNAc)_2_
12	1851.74	(Hex)_1_(HexNAc)_1_(Fuc)_1_ + (Man)_3_(GlcNAc)_2_
13	1867.73	(Hex)_2_(HexNAc_)1_ + (Man)_3_(GlcNAc)_2_
14	1892.76	(HexNAc)_2_(Fuc)_1_ + (Man)_3_(GlcNAc)_2_
15	1908.76	(Hex)_1_(HexNAc)_2_ + (Man)_3_(GlcNAc)_2_
16	1949.79	(HexNAc)_3_ + (Man)_3_(GlcNAc)_2_
17	1988.76	(Hex)_4_ + (Man)_3_(GlcNAc)_2_
18	2013.79	(Hex)_2_(HexNAc)_1_(Fuc)_1_ + (Man)_3_(GlcNAc)_2_
19	2029.79	(Hex)_3_(HexNAc)_1_ + (Man)_3_(GlcNAc)_2_
20	2054.82	(Hex)_1_(HexNAc)_2_(Fuc)_1_ + (Man)_3_(GlcNAc)_2_
21	2070.81	(Hex)_2_(HexNAc)_2_ + (Man)_3_(GlcNAc)_2_
22	2095.84	(HexNAc)_3_(Fuc)_1_ + (Man)_3_(GlcNAc)_2_
23	2111.84	(Hex)_1_(HexNAc)_3_ + (Man)_3_(GlcNAc)_2_
24	2150.81	(Hex)_5_ + (Man)_3_(GlcNAc)_2_
25	2152.87	(HexNAc)_4_ + (Man)_3_(GlcNAc)_2_
26	2175.84	(Hex)_3_(HexNAc)_1_(Fuc)_1_ + (Man)_3_(GlcNAc)_2_
27	2216.87	(Hex)_2_(HexNAc)_2_(Fuc)_1_ + (Man)_3_(GlcNAc)_2_
28	2232.87	(Hex)_3_(HexNAc)_2_ + (Man)_3_(GlcNAc)_2_
29	2257.90	(Hex)_1_(HexNAc)_3_(Fuc)_1_ + (Man)_3_(GlcNAc)_2_
30	2273.89	(Hex)2(HexNAc)3 + (Man)_3_(GlcNAc)_2_
31	2298.92	(HexNAc)_4_(Fuc)_1_ + (Man)_3_(GlcNAc)_2_
32	2312.86	(Hex)_6_ + (Man)_3_(GlcNAc)_2_
33	2314.92	(Hex)_1_(HexNAc)_4_ + (Man)_3_(GlcNAc)_2_
34	2359.93	(Hex)_1_(HexNAc)_2_(Fuc)_1_(NeuAc)_1_ + (Man)_3_(GlcNAc)_2_
35	2362.93	(Hex)_2_(HexNAc)_2_(Fuc)_2_ + (Man)_3_(GlcNAc)_2_
36	2375.93	(Hex)_2_(HexNAc)_2_(NeuAc)_1_ + (Man)_3_(GlcNAc)_2_
37	2378.92	(Hex)_3_(HexNAc)_2_(Fuc)_1_ + (Man)_3_(GlcNAc)_2_
38	2403.95	(Hex)_1_(HexNAc)_3_(Fuc)_2_ + (Man)_3_(GlcNAc)_2_
39	2419.95	(Hex)_2_(HexNAc)_3_(Fuc)_1_ + (Man)_3_(GlcNAc)_2_
40	2435.94	(Hex)_3_(HexNAc)_3_ + (Man)_3_(GlcNAc)_2_
41	2460.98	(Hex)_1_(HexNAc)_4_(Fuc)_1_ + (Man)_3_(GlcNAc)_2_
42	2474.92	(Hex)_7_ + (Man)_3_(GlcNAc)_2_
43	2476.97	(Hex)_2_(HexNAc)_4_ + (Man)_3_(GlcNAc)_2_
44	2521.99	(Hex)_2_(HexNAc)_2_(Fuc)_1_(NeuAc)_1_ + (Man)_3_(GlcNAc)_2_
45	2537.98	(Hex)_3_(HexNAc)_2_(NeuAc)_1_ + (Man)_3_(GlcNAc)_2_
46	2563.01	(Hex)_1_(HexNAc)_3_(Fuc)_1_(NeuAc)_1_ + (Man)_3_(GlcNAc)_2_
47	2566.01	(Hex)_2_(HexNAc)_3_(Fuc)_2_ + (Man)_3_(GlcNAc)_2_
48	2582.00	(Hex)_3_(HexNAc)_3_(Fuc)_1_ + (Man)_3_(GlcNAc)_2_
49	2623.03	(Hex)_2_(HexNAc)_4_(Fuc)_1_ + (Man)_3_(GlcNAc)_2_
50	2627.02	(Hex)_3_(HexNAc)_1_(Fuc)_2_(NeuAc)_1_ + (Man)_3_(GlcNAc)_2_
51	2639.02	(Hex)_3_(HexNAc)_4_ + (Man)_3_(GlcNAc)_2_
52	2725.06	(Hex)_2_(HexNAc)_3_(Fuc)_1_(NeuAc)_1_ + (Man)_3_(GlcNAc)_2_
53	2785.08	(Hex)_3_(HexNAc)_4_(Fuc)_1_ + (Man)_3_(GlcNAc)_2_
54	2799.02	(Hex)_9_ + (Man)_3_(GlcNAc)_2_
55	2827.10	(Hex)_2_(HexNAc)_2_(Fuc)_1_(NeuAc)_2_ + (Man)_3_(GlcNAc)_2_
56	2887.12	(Hex)_3_(HexNAc)_3_(Fuc)_1_(NeuAc)_1_ + (Man)_3_(GlcNAc)_2_
57	2947.13	(Hex)_4_(HexNAc)_4_(Fuc)_1_ + (Man)_3_(GlcNAc)_2_
58	2989.15	(Hex)_3_(HexNAc)_2_(Fuc)_1_(NeuAc)_2_ + (Man)_3_(GlcNAc)_2_
59	3252.25	(Hex)_4_(HexNAc)_4_(Fuc)_1_(NeuAc)_1_ + (Man)_3_(GlcNAc)_2_

### RGP binding to MGL/CD301 increases by trimming of *N*-glycans

**Figure 5:**
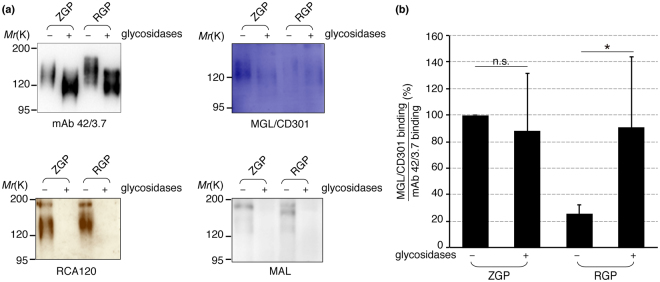
Binding of MGL/CD301 to RGP increased after glycosidases treatment. (**a**) Lysates of VLPs with RGP or ZGP were treated or untreated with sialidase and galactosidase, electrophoretically separated and subjected to immunoblotting with mAb 42/3.7, MGL/CD301, RCA-120, or MAL. The removal of sialic acid (NeuAc) and galactose by the enzymatic treatment was confirmed by MAL and RCA-120, respectively. Shown are cropped images from representative results (n = 4) and whole blot images are shown in Fig. S5. (**b**) The binding capacity of glysosidase-treated RGP/ZGP to MGL/CD301 was compared to that of untreated RGP/ZGP. Relative intensity of MGL/CD301 bindings to mAb 42/3.7 is shown (n = 4). In this quantification, relative intensity of untreated ZGP was set to 100%. For statistical analysis, Student’s *t*-test was performed. *p < 0.05, n.s.: not significant.

To check this hypothesis, VLPs bearing RGP were treated with neuraminidase and β-galactosidase, and the effect of *N*-glycan trimming on the interactions of GPs with MGL/CD301 was examined. The treated samples were separated by SDS-PAGE and bindings of mAb 42/3.7, *Maackia amurensis* leucoagglutinin (MAL), *Ricinus communis* agglutinin-120 (RCA-120), and recombinant MGL/CD301 were examined as shown in Fig. 5a. Bindings of mAb 42/3.7 revealed that the electrophoretic migration distance altered. Bindings of both MAL and RCA-120 completely diminished after treatment of RGP-bearing VLPs with these glycosidases, confirming the successful removal of sialic acid and galactose residues. The binding of MGL/CD301 to RGP increased after enzymatic treatments (Fig. 5a,b). These results strongly suggest that extension of *N*-glycans on RGP is responsible for the reduced interaction with MGL/CD301 and the lower infectivity of VSV pseudotyped with RGP compared to VSV pseudotyped with ZGP.

### *N*-terminal short peptide sequence regulates the extension pattern of *N*-glycosylation on GPs

To further confirm that the differential extention of *N*-glycans between RGP and ZGP was regulated by N-terminal amino acid residues 33–50 or 33–186 of GP, we analyzed the glycan extension pattern of chimeric GPs (Z33-50R, Z33-186R, R33-50Z, R33-186Z). As shown in Fig. 4c, the relative ratio of extended *N*-glycans increased by swapping the N-terminal residues 33–50 or 33–186 from ZGP into RGP (Z33-50R, Z33-186R). In contrast, the swapping of the *N*-terminal residues 33–50 or 33–186 from RGP into ZGP (R33-50Z, R33-186Z) resulted in the reduction of the relative ratio of extended-type *N*-glycans. These results strongly suggest that the short peptide sequence near the N-terminus of the GPs regulates the extension of *N*-glycans.

## Discussion

Glycan-lectin interactions play crucial roles in virus-host interactions, yet many of the underlying mechanisms have not been well-defined. In the present study, we focused on the interaction between glycans present on EBOV GPs and a C-type lectin, MGL/CD301 on the host cells, and aimed to answer the question why REBOV is less pathogenic than ZEBOV. We demonstrated that VSV pseudotyped with EBOV GP infected into DCs mediated by MGL/CD301 and that the N-terminal 33–50 amino acid sequence of GP determines the differential infectivity between VSV pseudotyped with RGP and ZGP. This differential infectivity seems to depend on the extension patterns of *N*-glycans on GPs, which in turn is regulated by the N-terminal 33–50 amino acid sequence of GP itself.

Other host cell molecules, including folate receptor-α^26^, Tyro3 family molecules^27^, T-cell immunoglobulin and mucin domain 1^28^, and other C-type lectins^12–18^ expressed on the initial target cells of filovirus (*e.g*. monocyte/macrophage, DCs, hepatocytes, sinusoidal endothelial cells) are reported to be involved in the initiation of infection. Those C-Type lectins, which were previously reported, have diverse carbohydrate specificities, for instance, DC-SIGN preferentially binds to mannose (Man). It is likely that C-type lectins with various carbohydrate specificities^29–32^ act in concert to facilitate the attachment and the entry of EBOV into DCs. MGL/CD301 seems to play predominant roles among these lectins and non-lectin receptors, because the MGL/CD301 blocking monoclonal antibody blocked the infection of pseudotyped viruses into human immature DCs by more than 60% (Fig. 1a).

The filovirus envelope GP, which is the only surface molecule present on viral particles, is known to be involved in virus entry into cells^2,5–11^. Manicassamy and co-workers reported that the *N*-terminal 150 amino acid residues of GP1 were important for the maintenance of protein integrity, the formation of a pseudotyped virus, and the viral entry into HEK293T cells, based on their single amino acid mutagenesis analysis^33^. They also claimed that several key amino-acid residues are crucial to the protein folding^33^. However, the importance of glycosylation of GP1 was not explored in their study. Marzi and co-workers reported that the signal sequence of GP (*N*-terminal 1–32 amino acid residues) affected the GP glycosylation that resulted in an enhancement of DC-SIGN/R-mediated viral infection^34^. They showed that high-mannose type glycosylation of SGP increased when the signal peptide of SGP was replaced with that of ZGP, though they did not study the glycan structures attached to GPs.

Our structural analyses by MS revealed that *N*-glycans of RGP are more extended than those of ZGP, even though these GPs were prepared in the same cell line (Fig. 4c). The extension patterns apparently affect the interaction with MGL/CD301and the binding of MGL/CD301 to RGP increased to a level to that to ZGP after the removal of *N*-glycans (Fig. 3d,e). The binding of MGL/CD301 to RGP was also increased when their extended glycans were trimmed using glycosidases (Fig. 5b). These results support that the structures of *N*-glycans of GPs affect the interaction between MGL/CD301 and GPs. More extended *N*-glycans of RGP seem to lower the binding to MGL/CD301, which preferentially binds *O*-glycans, and to decrease the capacity of GPs to mediate the infection of DCs and macrophages. *N*-glycans of ZGP do not seem to have such capacity (Fig. 3d,e).

Using a molecular genetic approach, we demonstrated that limited numbers of amino acid residues of GPs were critical for the differential lectin binding between RGP and ZGP. As far as we examined, a stretch of 18 amino acid residues near the amino terminus of the GP (33–50) seemed to play a key role. We also showed that the same stretch of amino acid residues plays an important role in the regulation of the differential *N*-glycan extension between RGP and ZGP. The lectin-binding potential and glycan extension pattern of ZGP and RGP were reversed when the *N*-terminal 33–50 (or 33–186) amino acid residues of the GPs were swapped (Figs 3a, 4d). These results strongly suggest that there is an oligopeptide motif that can control *N*-glycan extension. How this extension is controlled, however, remains to be determined. It is also worthwhile to mention that, in contrast to a previous report^34^, the signal sequence (1–32 amino acid residues) of GP did not seem to be involved in this phenomenon. We did not observe any effect of the signal sequence on the differential infectivity of pseudotyped viruses nor MGL/CD301 binding between REBOV and ZEBOV GPs (Fig. 2, ZGP vs Z33-462R, or Z1-462R vs Z33-462R).

Regarding the potential mechanism that controls *N*-glycan extension, we assumed that intracellular localization, trafficking speed, or trafficking pathway of the GP might be influenced by this peptide motif. However, as far as we examined, the biosynthetic rate and the intracellular localization of GP did not seem to be affected. Pulse-chase experiments on ^35^S-Methionine incorporation revealed almost identical biosynthetic rates between RGP and ZGP (Fig. S3a). The intracellular localizations of RGP and ZGP were very similar (Fig. S3b). We attempted to prepare VSV pseutotyped with RGP/ZGP lacking amino acid residues 33–50 (RGPΔ33-50, ZGPΔ33-50) and VLPs bearing RGPΔ33-50/ZGPΔ33-50 to confirm the importance of this oligopeptide motif. The expression of those GPs on VSV pseudotyped viruses or VLPs were confirmed by immunoblotting using mAb 42/3.7 (Fig. S6a,b), however, infectious viruses were not obtained (Fig. 6c) for unknown reasons. The band patterns of RGPΔ33-50 and ZGPΔ33-50 from VLPs were different from those of other GPs (Fig. S6b) suggesting that the amino acid residue 33rd through 50th of GP affect some processing of GPs. Further studies are needed to elucidate the mechanism by which this peptide sequence regulates *N*-glycan extension.

Collar and co-workers recently reported results of structural analyses of *N*- and *O*-glycans of recombinant EBOV GPs^35^. They concluded by comparing *Zaire ebolavirus, Sudan ebolavirus, Bundibugyo ebolavirus, Tai Forest ebolavirus* that *N*-glycan compositions were similar but *O*-glycan structures varied. However, in contrast to our study, they analyzed recombinant GPs from the lysates of 293 T cells expressing GPs, while our study was on GPs from VLPs which closely represent viral particles. Furthermore, they did not analyze RGP which represent the molecular characteristics of a poorly infectious variant. Our results on the *N*-glycan structures, however, were consistent with their results in that most of the *N*-glycans were fucosylated and asialo-type glycans (Fig. S4)^35^. According to their results, ZGP and SGP contained lower amounts of *O*-glycans with sialic acid than GPs of *Bundibugyo*/*Ivory Coast*. Further studies are necessary to prove or disprove the hypothesis that decreased sialylations contribute to elevated interactions of these GPs with MGL/CD301.

Previous reports showed that GP was useful as a therapeutic target for EBOV infection. GP-specific neutralizing antibodies protect experimental animals from lethal EBOV infection. Some antibodies were able to protect mice completely and guinea pigs partially^8,10^. Another neutralizing antibody was effective for guinea pigs but not for non-human primates^36,37^. Recently, several neutralizing antibodies that can protect macaques from lethal EBOV infection were also reported^38–40^. As shown in the present report, by enhancing *N*-glycan extension in cells infected with EBOV, infectivity of the virus produced by these cells may be reduced. Furthermore, structural analysis of GPs with extended *N*-glycans might assist to identify an epitope that could be used as a target of an effective therapeutic antibody against EBOV. Blockade of the infection should also be achieved by interfering with the interaction between GPs and MGL/CD301. The present study will also contribute to understand novel regulatory mechanisms of glycosylation in cells during infection.

## Materials and Methods

### Construction of chimeric GPs

Standard molecular biology technique was used to construct chimeric GPs containing pCAGGS vectors. The sequence of ZGP and RGP were obtained from MP1153-ZGP vector and pBluescript-RGP vetor respectively, and primers as described in Supplementary Table 1 were used.

### Generation of recombinant VSVΔG*-pseudotyped virus

VSVΔG*-pseudotyped viruses were generated as described previously with some modifications^5^. In the VSVΔG* genome, the coding region of the VSV G protein gene was replaced with the coding region of a modified version of the GFP gene^5^. GPs of ZEBOV (Strain Mayinga), REBOV (Strain Pennsylvania), SEBOV (Strain Boniface), and chimeric GPs were cloned into pCAGGS vector. These plasmids were transfected into human embryonic kidney (HEK293T) cells by using Trans-IT LT1 (Takara). Twenty-four hours after transfection, cells were infected with VSVΔG*-VSVG (VSV pseudotyped with VSVG) at a multiplicity of infection (MOI) of 1 for 1 hour at 37 °C. Cells were then washed twice with Dulbecco’s modified Eagle Medium-High Glucose (DMEM-HG) without fetal calf serum (FCS) and once with DMEM-HG with 10% FCS, and then medium was added. After 24 hours of incubation at 37 °C in a 5% CO_2_ atmosphere, the culture fluid was collected and centrifuged to remove cells. Each virus stock was stored at −80 °C until further use. Regarding to the biosafety level (BSL), the pseudotyped virus was categorized to BSL2.

### Production of Virus-like particles (VLPs)

VLPs were produced as reported previously with some modification^19,20^. Briefly, HEK293T cells (4 × 10^6^) were seeded on a 10 cm culture dish one day before transfection. These cells were transfected with pCAGGS-GP and pCAGGS-VP40 by using Trans-IT LT1. DNA and transfection reagent (40 μL of Trans-IT LT1 with 10 μg of each plasmid) were mixed in 0.5 mL of Opti-MEM (Gibco) and added to the cells. The mixture was incubated at 37 °C for 48 hours. The culture medium was harvested 48 hours later and centrifuged at 6,000 rpm for 20 min to remove cellular debris. The supernatant was then layered on a 25% sucrose cushion and ultra-centrifuged at 27,000 rpm for 1.75 hours at 4 °C using a himac CP 70 MX (Hitachi). The supernatant was discarded, and the pellet was resuspended in PBS. The suspension was regarded as VLPs and stored at −20 °C until further use. The protein amount of VLP solution was measured by BCA protein assay kit (Pierce).

### Cell culture

HEK293T cells and K562 human chronic myelogenous leukemia cells were grown in DMEM-HG or RPMI 1640 medium, respectively, supplemented with 10% FCS. Peripheral blood mononuclear cells (PBMCs) were isolated from venous blood drawn from healthy volunteers at the Tokyo Metropolitan Red Cross Blood Center (Tokyo, Japan) by centrifugation on a Percoll density gradient. Monocytes were purified from PBMCs by incubation with anti-CD14 mAb-coated microbeads and separation by a magnetic cell separation system (MACS, Miltenyi Biotec, Bergisch Gladbach, Germany) with column type LS. Immature DCs were prepared by culturing monocytes for 7 days in RPMI containing 7.5% FCS with 500 U/ml GM-CSF (Kyowa-Kirin) and 100 ng/mL interleukin-4 (GT #2204). To examine the purity of immature DCs, flow cytometric analysis with fluorescein isothiocyanate (FITC)-labeled anti-human CD1a (Biosource), CD14 (Dako Cytomation), CD83 (eBioscience), and HLA-DR (BD Biosciences Pharmingen) was performed.

### Expression of soluble recombinant MGL/CD301

A plasmid encoding the putative extracellular domain of MGL/CD301 was prepared as described previously^32^. *E. coli* with expressed proteins were recovered, first washed with TBS containing 0.5% TritonX-100 (Sigma) and 10 mM EDTA, and then washed with H_2_O. Washed pellets were solubilized with 2 M NH_4_OH, and soluble recombinant MGL/CD301 was refolded in a mixture of 2 mM reduced glutathione and 0.2 mM oxidized glutathione. The refolded protein was dialyzed against 20 mM MOPS buffer (pH 7.0) containing 20 mM CaCl_2_, 0.5 M NaCl, and 0.02% NaN_3_ at 4 °C. Soluble rMGL/CD301 was purified by affinity chromatography on a galactose-Sepharose 4B column.

### Transfection and expression of human MGL/CD301 cDNA in K562 cells

The coding sequence of MGL/CD301 was inserted into a mammalian cell expression vector, pRc/CMV (Invitrogen), in both sense and anti-sense orientations. K562 cells were transfected with the plasmids by electroporation. After selection with Geneticin (G418 sulfate; Gibco), MGL/CD301-positive cells were enriched with immunomagnetic beads by using an anti-MGL/CD301 monoclonal antibody (mAb) MLD-1. Cloning of transfectants was performed by the limiting dilution method. Flow cytometric analysis with mAb MLD-1 (1:400-diluted ascites fluid) and FITC-labeled anti-mouse immunoglobulin G (Zymed, 5 μg/mL) was performed to determine the levels of MGL/CD301 expression on the obtained cell clones.

### Titration of VSVΔG*-pseudotyped virus

Vero E6 cells (5 × 10^4^ cells/well) were seeded on 96-well plate one day before the infection. Serially diluted viral solutions were made after treatment with anti-VSVG antibody at room temperature for 30 min to neutralize VSV pseudotyped with VSVG in the viral solution, and diluted viral solution was added 50 μL/well to seeded cells. Cells were incubated at 37 °C for 16 h, then the number of infected cells were analyzed by flow cytometory. In our infection experiment, virus titer was defined as below. Titer = {(number of infected cells) × (dilution factor of viral stock)} ÷ (volume of viral solution).

### Infection experiment

VSV pseudotyped with GPs was incubated with monocyte-derived dendritic cells or with K562-MGL/CD301 cells and incubated for 48 hours. The numbers of infected cells were determined by counting GFP-positive cells by flow cytometry. When K562-MGL/CD301 cells were used as the target, the percentage of infected cells was calculated relative to the number of infected K562-mock cells. The inoculum dose of each virus was calculated based on the according to the following formula. Inoculum required (mL) = {MOI* × (number of cells)} ÷ titer, *MOI; multiplicity of infection, MOI = 0.05~1.

### SDS-PAGE and immunoblotting analysis

VSV pseudotyped with GPs or VLPs bearing GP were solubilized. Viral GPs were separated by SDS-PAGE and blotted onto PVDF membrane. The membrane was blocked with 3% BSA in PBS at 4 °C overnight, and incubated with anti-EBOV GP1 mAb 42/3.7 (1:1000 dilution), followed by HRP goat anti-mouse IgG(H + L) (ZyMed) to confirm equal loading. Biotinylated lectins (2.5 μg/ml in TBS containing Ca^2+^) were reacted for 2 hours at room temperature, and then ZyMax^TM^ streptavidin alkaline phosphatase conjugate (Zymed, 1:1000 dilution) was used as the second reagent for 1 hour at room temperature. After washing, the membranes were developed using ECL^TM^ Western blotting detection reagent (Amersham) for HRP or alkaline phosphatase substrate Kit IV (Vector) for alkaline phosphatase. Band intensities of the MGL/CD301 binding and the anti-EBOV GP1 mAb 42/3.7 binding to the GPs was quantified with Image J or ImageQuant TL (GE Healthcare) software. In the measurement of MGL/CD301 binding to GPs, the amounts of GPs were normalized as stated below. First, the loading amounts of viral lysates or VLPs were normalized to the total protein amount measured using BCA protein assays. Western blotting analysis was performed using mAb 42/3.7. Bands intensities of GPs were quantified by ImageJ or ImageQuant TL software, and loading volume of viral lysate or VLPs were re-normalized to load the estimated equal amount of GPs.

### PNGase F treatment

One µg of VLPs bearing ZGP or RGP were treated with 1.5 units of PNGase F (Roche) in a reaction mixture containing 5 mM EDTA (pH 8.0), 1% 2-mercaptoethanol (2-ME), and 40 mM Tris-HCl buffer (pH 8.5) at 37 °C for 18 hours. Viral GPs from VLPs were electrophoretically separated, blotted onto PVDF membranes and analyzed by immunoblotting.

### Structural estimation of *N*-glycans

Immunoprecipitated GPs from solubilized VLPs with GPs were separated on an 8% SDS-PAGE gel. The gel was then fixed and stained with CBB (BioRad). The stained portions of GPs in the gel were excised and subjected to in-gel tryptic digestion. Digestion was carried out for 16 h with MS-grade trypsin (Wako). Peptides were extracted from the gel with 0.1% TFA (containing 50% acetonitrile (ACN)). Peptides were dried by speed vac and dissolved in H_2_O. Dissolved peptide samples were treated with PNGase F (Roche) to release *N*-glycans. Released *N*-glycans were purified and labeled using the Glycoblotting method (Sumitomo Bakelite). Glycoblotting was performed according to a previously described procedure^25^. Labeled *N*-glycans were analyzed by MALDI-TOF MS and the TOF/TOF method using UltraFLEX II (Bruker).

### Glycosidase treatment

VLPs bearing ZGP or RGP were treated with 100 mU of Neuraminidase [*Clostridium perfringens*] (Sigma) at 37 °C for 2 hours. Then, 3 mU of β-galactosidase [*Streptococcus*] (Seikagaku Co.) and MnCl_2_ (final concentration: 10 mM) were added and incubated at 37 °C for 24 hours. Glycosidase-treated VLPs were subjected to immunoblotting to confirm the removal of NeuAc and Gal as well as the changes of MGL/CD301 binding before and after enzyme treatment.

### Statistical analysis

Student’s paired *t*-test was used to determine the statistical significance of the differences. A *p*-value of less than 0.05 was considered statistically significant.

## Electronic supplementary material

Supplementary Information
