# Supplementary material for: A Critical Domain of Ebolavirus Envelope Glycoprotein Determines Glycoform and Infectivity
Source: Sci Rep. 2018 Apr 3;8:5495. doi: 10.1038/s41598-018-23357-8 (PMC5882653; doi:10.1038/s41598-018-23357-8)
Supplement: Supplementary file 1 — Supplementary Information [file 41598_2018_23357_MOESM1_ESM.pdf]

# Supplementary Information

**Title: A Critical Domain of Ebolavirus Envelope Glycoprotein Determines Glycoform and Infectivity**

**Authors and Affiliations**

Haruhiko Fujihira[1,2,3*], Katsuaki Usami[1], Keita Matsuno[4,5], Hideyuki Takeuchi[1,6], Kaori Denda-Nagai[1,2], Jun-ichi Furukawa[7,8], Yasuro Shinohara[7,9], Ayato Takada[5,10], Yoshihiro Kawaoka[11,12,13], and Tatsuro Irimura[1,2*]

[1]Laboratory of Cancer Biology and Molecular Immunology, Graduate School of Pharmaceutical Sciences, The University of Tokyo, Tokyo 113-0033, Japan

[2]Division of Glycobiologics, Intractable Disease Research Center, Juntendo University Graduate School of Medicine, Tokyo 113-8421, Japan

[3]Glycometabolome Team, Systems Glycobiology Research Group, Global Research Cluster, RIKEN, Saitama 351-0198, Japan

[4]Laboratory of Microbiology, Faculty of Veterinary Medicine, Hokkaido University, Sapporo 060-0818, Japan

[5]Division of International Services, Global Institution for Collaborative Research and Education (GI-CoRE), Hokkaido University, Sapporo 001-0020, Japan

[6]Department of Molecular Biochemistry, Nagoya University School of Medicine, Nagoya 4668550, Japan

[7]Laboratory of Medical and Functional Glycomics, Graduate School of Advanced Life Science, Hokkaido University, Sapporo 001-0021, Japan

[8]Department of Advanced clinical glycobiology, Faculty of Medicine and Graduate School of Medicine, Hokkaido University, Sapporo 0010021, Japan

[9]Department of Pharmacy, Kinjo Gakuin University, Nagoya 4638521, Japan

[10]Division of Global Epidemiology, Hokkaido University Research Center for Zoonosis Control, Sapporo 001-0020, Japan
[11]CREST, Japan Science and Technology Agency, Saitama 332-0012, Japan
[12]Division of Virology, Department of Microbiology and Immunology, Institute of Medical Science, The University of Tokyo, Tokyo 108-8639, Japan
[13]Department of Pathobiological Sciences, University of Wisconsin, Madison, WI 53706, USA

**Supplementary Figure Legends**

**Supplementary Figure 1. Amino acid sequences of ZGP and RGP.** Amino acid sequences of ZGP and RGP are shown. Red box: 33-50 amino acid residue, green box: mucin-like domain, orange line: border of GP1 and GP2.

**Supplemental Figure 2. MALDI-TOF MS spectra of *N*-glycans released from VLPs.** Representative MS spectra of *N*-glycans released from VLPs bearing **(a)** ZGP, **(b)** RGP, **(c)** Z33-50R, **(d)** Z33-186, **(e)** R33-50, or **(f)** R33-186 are shown. Magnified spectra between m/z=3800 and m/z=4200 are shown in the upper right window of each spectrum.

**Supplementary Figure 3. Biosynthetic rate and intracellular localization of RGP and ZGP were not different.** **(a)** Results of pulse chase experiments of [$^{35}$S]-labeled

RGP and [$^{35}$S]-labeled ZGP are shown. **(b)** Results of immunofluorescent staining of VLP-producing HEK293T cells. Red: GP (ZGP or RGP), Green: Golgi marker (GM130 or TGN46). Representative results are shown.

**Supplementary Figure 4. Sialylation and fucosylation of *N*-glycans found in GPs were not different.** Relative ratios of sialylated **(a)** or fucosylated **(b)** *N*-glycans are shown.

**Supplementary Figure 5. Uncropped images of blots shown in the figures (Fig. 1c, 1d, 2b, 3b, 3d, 5a).**

**Supplementary Figure 6. GPs that lack 18 amino acid residues (33 through 50) is expressed on the VSV pseudotyped viruses and VLPs.**

**(a-b)** Results of immunoblotting using VSV pseudotyped viruses or VLPs containing ZGPΔ33-50 or RGPΔ33-50 are shown. **(c)** Results of virus titration experiment of VSV pseudotyped ZGP, RGP, ZGPΔ33-50, and RGPΔ33-50 using Vero E6 cells. VSV pseudotyped with ZGP/RGP without 18 amino acids (N-terminal 33rd through 50th) did not show any infectivity to Vero E6 cells. (n=3)

**Supplementary Materials and Methods**

**Pulse chase experiment.** HEK293T cells ($6\times10^5$) were seeded on a 6-well culture plate one day before transfection. Cells were transfected with pCAGGS-GP and pCAGGS-VP40 by using Trans-IT LT1 in the same way as in VLP production. The cells were washed with PBS 24 hours after transfection and subsequently cultured with DMEM-HG (10% FCS, without Met and Cys; Gibco) for 1 hour. Then the medium was changed to DMEM-HG (10% FCS, containing 150 μCi/mL of [$^{35}$S]-labeled Met and Cys) and cultured for 30 min. The medium was changed to DMED-HG (10% FCS) after washing with DMEM-HG (10% FCS) and cultured for 0, 1, 2, or 3 hours. Cells were collected after washing with PBS and solubilized with RIPA buffer containing protease inhibitor cocktail (Calbiochem) by shaking at 4°C for 2 hours. The solution was centrifuged at 21,500*g* for 15 min to remove cellular debris. The supernatant was collected, and the radioactivity of [$^{35}$S] in the supernatant was measured by a liquid scintillation counter (LS6000SC, BECKMAN). GPs were immunoprecipitated using mAb 42/3.7 from lysates containing equal amounts of [$^{35}$S] radioactivity. Immunoprecipitated GPs were separated on an 8% SDS-PAGE gel. The gel was then

fixed and stained with CBB (BioRad). The gel was dried (RapiDry mini; ATTO) and [$^{35}$S]-labeled GPs were detected with Typhoon FLA9000 (GE Healthcare).

**Immunofluorescent staining.** HEK293T cells (4×10$^{6}$) were seeded on a 10 cm culture dish one day before transfection. Cells were transfected with pCAGGS-GP and pCAGGS-VP40 by using Trans-IT LT1 in the same way as in VLP production. 24 hours after transfection, cells were washed with PBS three times and collected. Collected cells were fixed with 20 mM sodium phosphate buffer (pH 7.0) containing 4 (w/v) % paraformaldehyde at 4°C for 1 hour. Fixed cells were washed with 10 mM glycine in PBS, and permeabilized by 0.1 (w/v) % Triton X-100 in PBS (at room temperature for 5 min). The cells were blocked with 4 (w/v) % BSA in PBS at room temperature for 30 min, and incubated with anti-EBOV GP1 mAb 42/3.7 (10 μg/mL) and rabbit anti-GM130 (10 μg/mL; Abcam) or rabbit anti-TGN46 antibody (10 μg/mL; Abcam), followed by Alexa 568 goat anti-mouse IgG(H+L) (10 μg/mL; Molecular Probes) and Alexa 488 goat anti-rabbit IgG(H+L) (10 μg/mL; Molecular Probes) and DAPI (Boehringer Mannheim). Analysis was performed using a Leica TSC SP5 confocal microscope (Leica).

**Supplemental Table 1. Primer sequences that used to make chimeric GPs**

| Chimeric GPs | | Primer Sequences |
|---|---|---|
| Z311-462R | forward | CCGCTCGAGGTCTCGATAGAACTGTGAAAGACAACTCTTC |
| | reverse | CGGAATTCGGTCTCCAACACTCATCACCAAGATACC |
| Z297-462R | forward | CCGAATTCGGTCTCGAAAAGTTTTTTTAGTTTCCCAGAAGGC |
| | reverse | CGGAATTCGGTCTCCAACACTCATCACCAAGATACC |
| Z260-462R | forward | CCGAATTCGGTCTCCGAAGTGTCTCATTCAGCTGGAGC |
| | reverse | CGGAATTCGGTCTCCAACACTCATCACCAAGATACC |
| Z187-462R | forward | CCGAATTCGGTCTCTCTGACAGTATCAGAAATGCAACGAC |
| | reverse | CGGAATTCGGTCTCCAACACTCATCACCAAGATACC |
| Z33-462R | forward | CGCGGTCTCGGCATGGAAAATGTTCTTTGGAAAAGG |
| | reverse | CGGAATTCGGTCTCCAACACTCATCACCAAGATACC |
| Z1-462R | forward | CCGCTCGAGGTCTCCCCATTGTGTTGTTGGATCCTC |
| | reverse | CGGAATTCGGTCTCCAACACTCATCACCAAGATACC |
| Z33-186R | forward | CGCGGTCTCCATGCCGCTTGGTATAGTG |
| | reverse | CGCGGTCTCCGGGGCAGAATTAAAAAAGCTACGACACC |
| Z33-50R | forward | CGCGGTCTCCATGCCGCTTGGTATAGTG |
| | reverse | CGCGGTCTCACTAGTTGATCAATTTCTGTTGCTTTG |
| R33-186Z | forward | CGCGGTCTCCATCCCACTTGGAGTCATC |
| | reverse | CGCGGTCTCTCTGACAGTATCAGAAATGCAACGACA |
| R33-50R | forward | CGCGGTCTCACTAGTTTGTCGTGACAAAC |
| | reverse | CGCGGTCTCGGCATGGAAAATGTTCTTTGGAAAAGG |

Figure S1

```
ZGP   1 MGVT-GILQL PRDRFKRTSF FLWVIILFQR TFSIPLGVIH NSTLQVSDVD
        **     *** ** **  ***   ********   * ***    ****     *
RGP   1 MGSGYQLLQL PRERFRKTSF LVWVIILFQR AISMPLGIVT NSTLKATEID

ZGP  50 KLVCRDKLSS TNQLRSVGLN LEGNGVATDV PSATKRWGFR SGVPPKVVNY
         ********* * ** ***** ***** **** ********** ******** *
RGP  50 QLVCRDKLSS TSQLKSVGLN LEGNGIATDV PSATKRWGFR SGVPPKVVSY

ZGP 100 EAGEWAENCY NLEIKKPDGS ECLPAAPDGI RGFPRCRYVH KVSGTGPCAG
        ********** ****** *** ****  ***  ********** ** ***** *
RGP 100 EAGEWAENCY NLEIKK DGS ECLPLPPDGV RGFPRCRYVH KVQGTGPCPG

ZGP 150 DFAFHKEGAF FLYDRLASTV IYRGTTFAEG VVAFLILPQA KKDFFSSHPL
        * ******** ********** ********** *******    ** *    *
RGP 150 DLAFHKNGAF FLYDRLASTV IYRGTTFAEG VVAFLILSEP KKHFWKATPA

ZGP 200 REPVNATEDP SSGYYSTTIR YQATGFGTNE TEYLFEVDNL TYVQLESRFT
         **** * *   * *   *   *    ** **    ** ***  *****    *
RGP 200 HEPVNTTDDS TSYYMTLTLS YEMSNFGGNE SNTLFKVDNH TYVQLDRPHT

ZGP 250 PQFLLQLNET IYTSGKRSNT TGKLIWKVNP EIDTTIGEWA FWETKKNLTR
        **** *****        **  ** * *   *  *    **** *******
RGP 250 PQFLVQLNET LRRNNRLSNS TGRLTWTLDP KIEPDVGEWA FWETKKNFSQ

ZGP 300 KIRSEELSFT VVSNGAKNIS GQSPARTSSD PGTNTTTEDH KIMASENSSA
            * * *    *    * *  **** *                     *
RGP 300 QLHGENLHFQ ILSTHTNNSS DQSPAGTVQG KISYHPPANN SELVPTDSPP

ZGP 350 MVQVHSQGRE AAVSHLTTLA TISTSPQSLT TKPGPDNSTH NTPVYKLDIS
         * *   **     *    *     *     *   *          *
RGP 350 VVSVLTAGRT EEMST-QGLT NGETIT-GFT ANPMTTTIAP -SPTMTSEVD

ZGP 400 EATQVEQHHR RTD-NDSTAS DTPSAT--TA AGPPKAENTN TSKSTDFLDP
             **         **  *              *    *           *
RGP 400 NNVPSEQPNN TASIEDSPPS ASNETIYHSE MDPIQGSNNS AQSPQTKTTP

ZGP 447 ATTTSPQNHS ETAGNNNTHH QDTGEESASS GKLGLITNTI AGVAGLITGG
        * ****          *                *     **  **     **
RGP 447 APTTSPMTQD PQETANSSKP GTSPGSAAGP SQPGLTINTV SKVADSLSPT

ZGP 497 RRTRR  EAI VNAQPKCNPN LHYWTTQDEG AAIGLAWIPY FGPAAEGIYI
        *   *       *   ****  * ***  *** ** ******* **********
RGP 497 RKQKR  SVR QNTANKCNPD LYYWTAVDEG AAVGLAWIPY FGPAAEGIYI

ZGP 545 EGLMHNQDGL ICGLRQLANE TTQALQLFLR ATTELRTFSI LNRKAIDFLL
        ** **** ** ********** ********** ******* *  **********
RGP 545 EGVMHNQNGL ICGLRQLANE TTQALQLFLR ATTELRTYSL LNRKAIDFLL

ZGP 595 QRWGGTCHIL GPDCCIEPHD WTKNITDKID QIIHDFVDKT LPDQGDNDNW
        ******* ** ** ******* ******* *  ** *** *   *** **  *
RGP 595 QRWGGTCRIL GPSCCIEPHD WTKNITDEIN QIKHDFIDNP LPDHGDDLNL

ZGP 645 WTGWRQWIPA GIGVTGVIIA VIALFCICKF VF
        ********** ***  *****  *** ****
RGP 645 WTGWRQWIPA GIGIIGVIIA IIALLCICKI LC
```

Figure S2

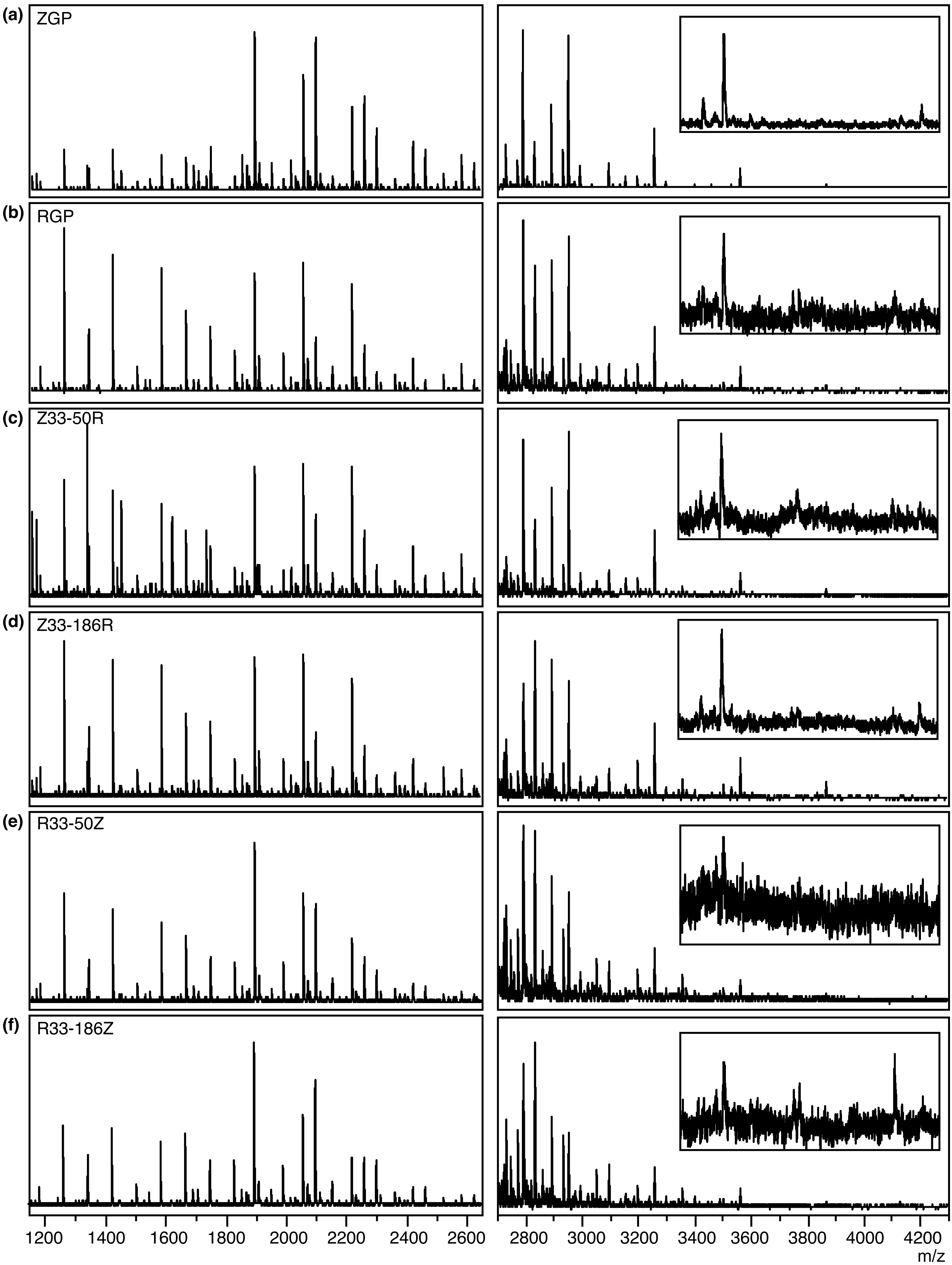

(a)
ZGP
(b)
RGP
(c)
Z33-50R
(d)
Z33-186R
(e)
R33-50Z
(f)
R33-186Z
1200
1400
1600
1800
2000
2200
2400
2600
2800
3000
3200
3400
3600
3800
4000
4200
m/z

Figure S3

(a)

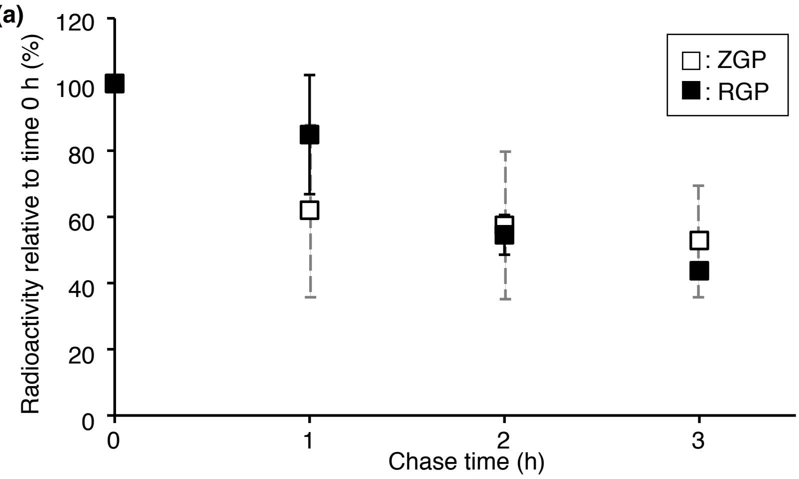

Radioactivity relative to time 0 h (%)
120
100
80
60
40
20
0
0
1
2
3
Chase time (h)
□: ZGP
■: RGP


(b)

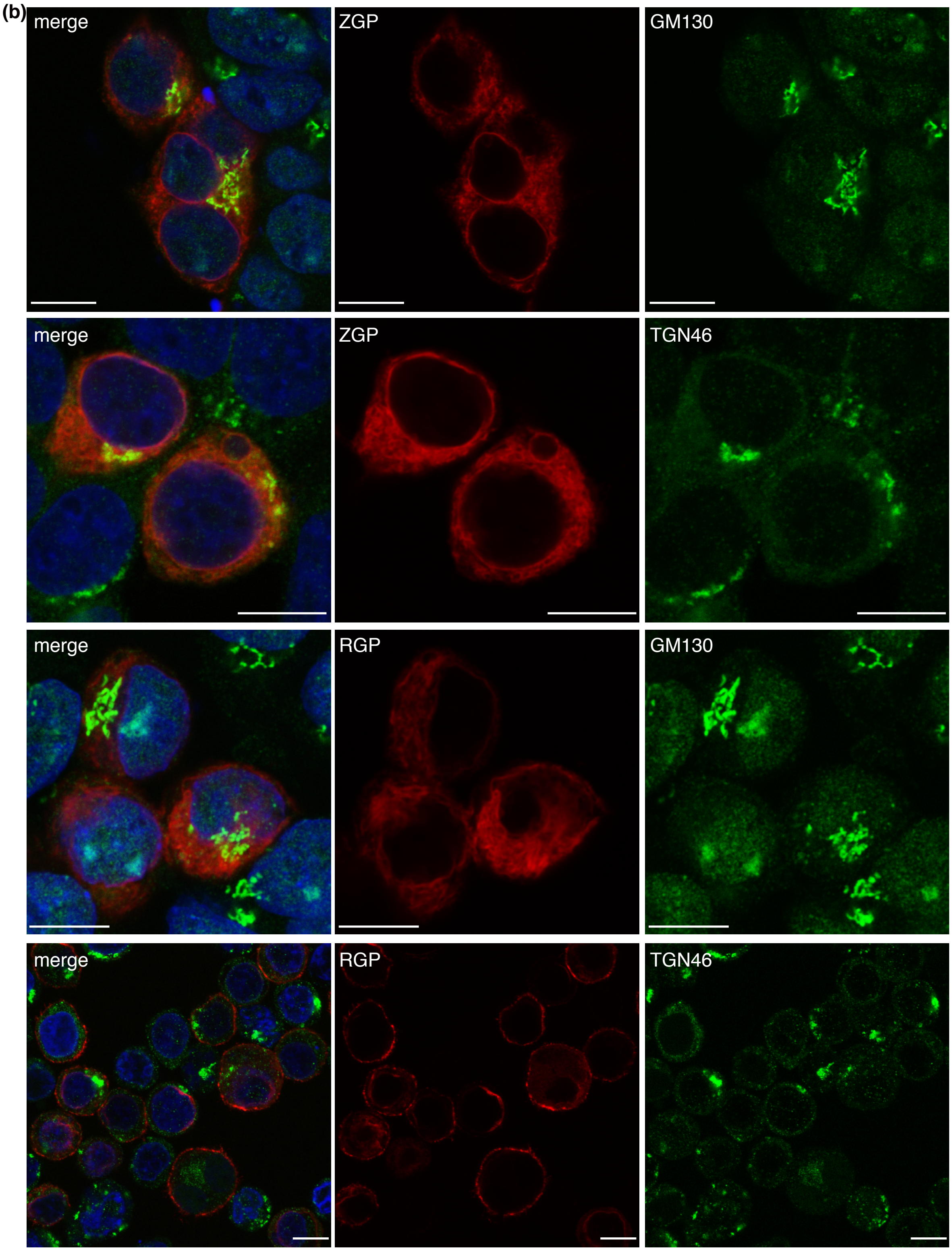

merge
ZGP
GM130
merge
ZGP
TGN46
merge
RGP
GM130
merge
RGP
TGN46

Figure S4

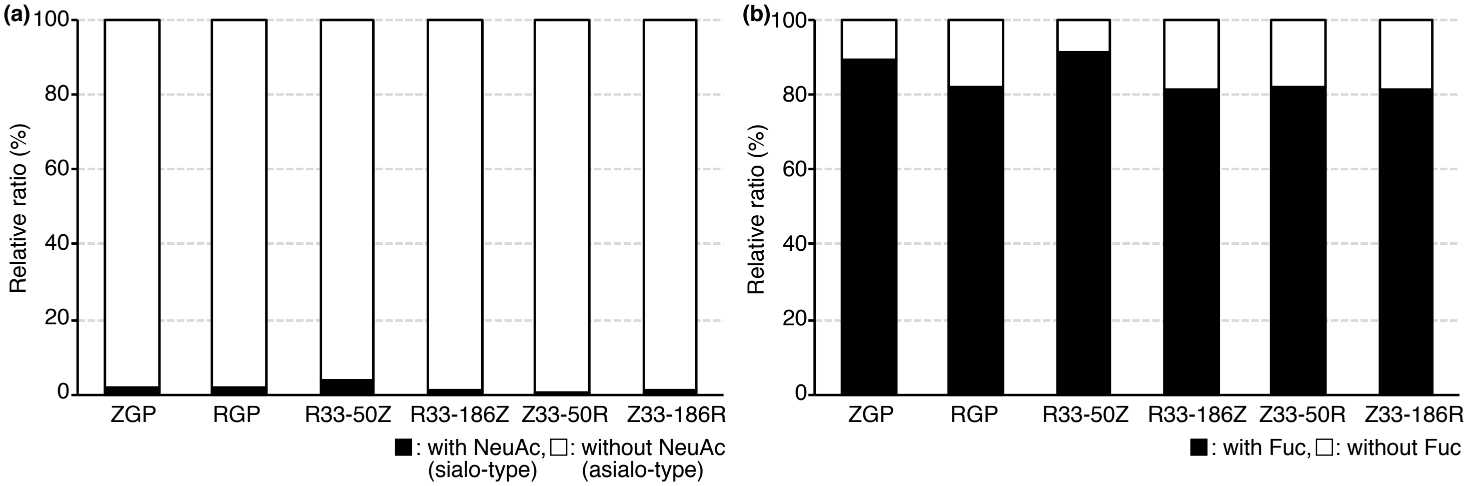

(a)
Relative ratio (%)
100
80
60
40
20
0
ZGP
RGP
R33-50Z
R33-186Z
Z33-50R
Z33-186R
■: with NeuAc, □: without NeuAc
(sialo-type) (asialo-type)
(b)
Relative ratio (%)
100
80
60
40
20
0
ZGP
RGP
R33-50Z
R33-186Z
Z33-50R
Z33-186R
■: with Fuc, □: without Fuc

Figure S5

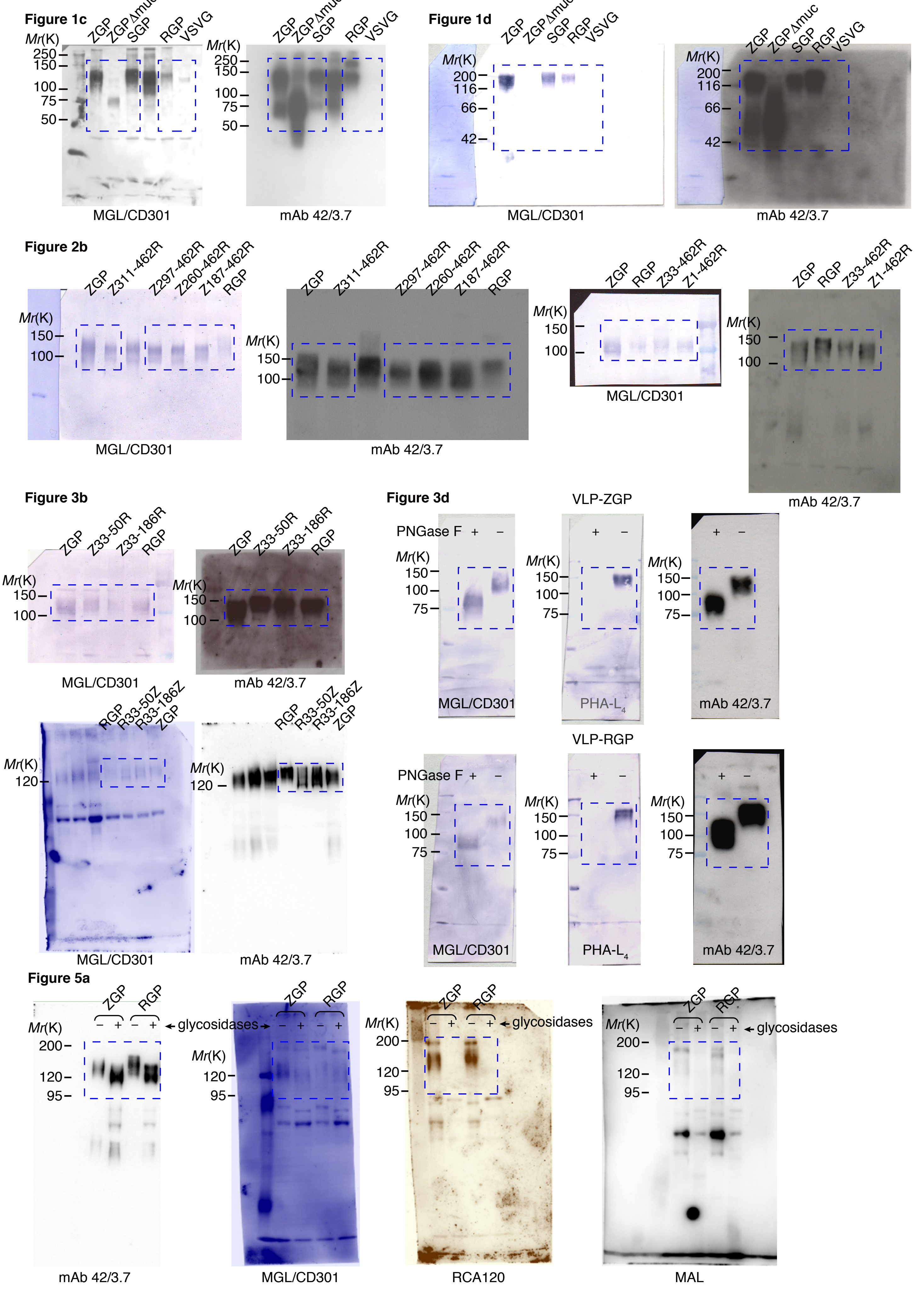
Figure 1c
ZGP ZGPΔmuc SGP RGP VSVG
Mr(K)
250
150
100
75
50
MGL/CD301
mAb 42/3.7
Figure 1d
ZGP ZGPΔmuc SGP RGP VSVG
200
116
66
42
MGL/CD301
mAb 42/3.7
Figure 2b
ZGP Z311-462R Z297-462R Z260-462R Z187-462R RGP
MGL/CD301
mAb 42/3.7
ZGP RGP Z33-462R Z1-462R
MGL/CD301
mAb 42/3.7
Figure 3b
ZGP Z33-50R Z33-186R RGP
MGL/CD301
mAb 42/3.7
RGP R33-50Z R33-186Z ZGP
120
MGL/CD301
mAb 42/3.7
Figure 3d
VLP-ZGP
PNGase F + –
MGL/CD301
PHA-L4
mAb 42/3.7
VLP-RGP
Figure 5a
ZGP RGP
glycosidases
95
mAb 42/3.7
MGL/CD301
RCA120
MAL

Figure S6

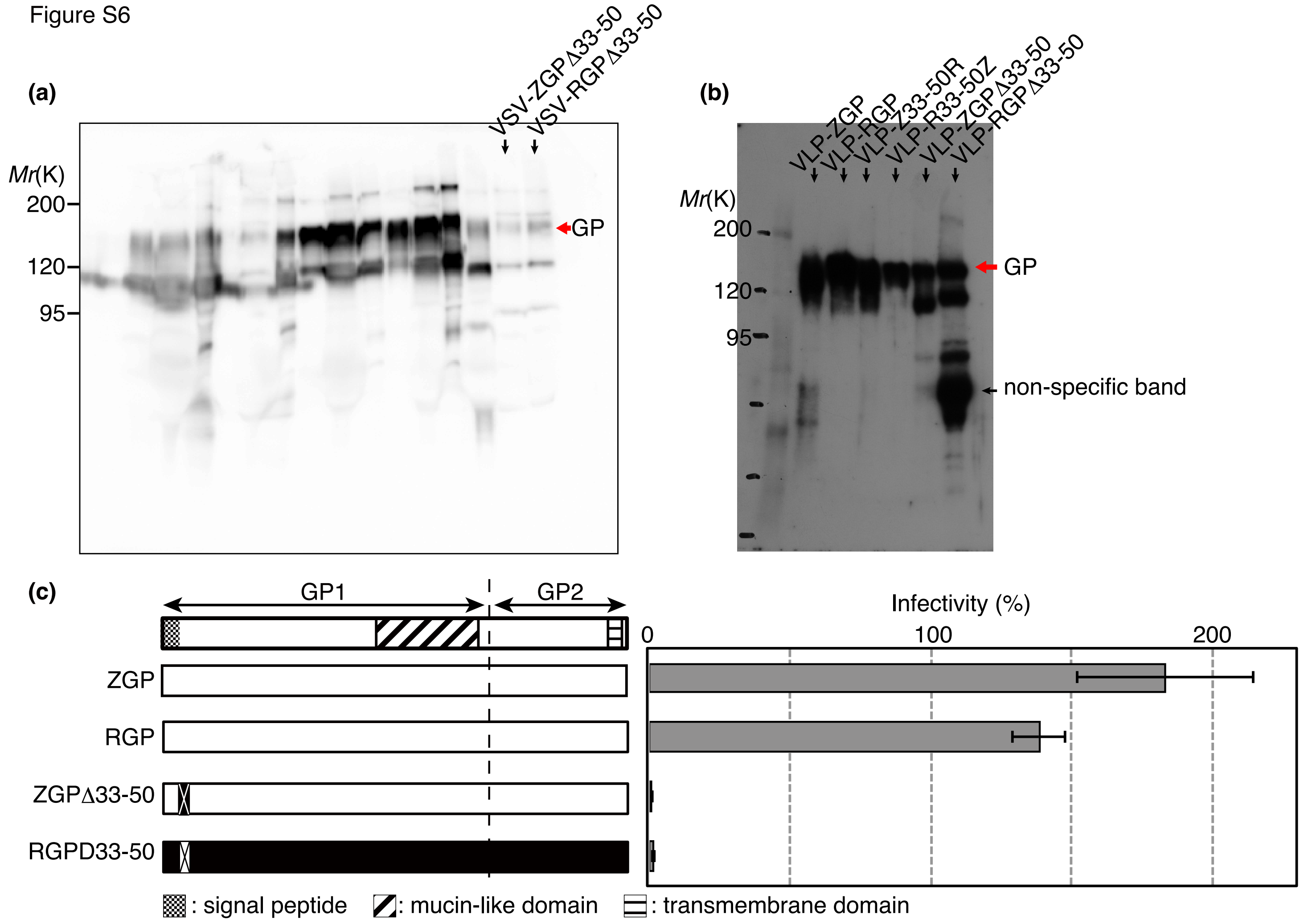
(a)
VSV-ZGPΔ33-50
VSV-RGPΔ33-50
Mr(K)
200
120
95
GP
(b)
VLP-ZGP
VLP-RGP
VLP-Z33-50R
VLP-R33-50Z
VLP-ZGPΔ33-50
VLP-RGPΔ33-50
Mr(K)
200
120
95
GP
non-specific band
(c)
GP1
GP2
ZGP
RGP
ZGPΔ33-50
RGPD33-50
Infectivity (%)
0
100
200
: signal peptide
: mucin-like domain
: transmembrane domain
